# Effect of ZnO and SnO_2_ Nanolayers at Grain Boundaries on Thermoelectric Properties of Polycrystalline Skutterudites

**DOI:** 10.3390/nano10112270

**Published:** 2020-11-16

**Authors:** Sang-il Kim, Jiwoo An, Woo-Jae Lee, Se Hun Kwon, Woo Hyun Nam, Nguyen Van Du, Jong-Min Oh, Sang-Mo Koo, Jung Young Cho, Weon Ho Shin

**Affiliations:** 1 Department of Materials Science and Engineering, University of Seoul, Seoul 02504, Korea; sang1.kim@uos.ac.kr (S.-i.K.); ajwoo8020@uos.ac.kr (J.A.); 2 School of Materials Science and Engineering, Pusan National University, Busan 46241, Korea; woojl@pusan.ac.kr (W.-J.L.); sehun@pusan.ac.kr (S.H.K.); 3 Energy & Environment Division, Korea Institute of Ceramic Engineering & Technology, Jinju 52851, Korea; whnam@kicet.re.kr (W.H.N.); du.nguyenvan@phenikaa-uni.edu.vn (N.V.D.); 4 Faculty of Materials Science and Engineering, Phenikaa University, Yen Nghia, Ha-Dong District, Hanoi 10000, Vietnam; 5 Department of Electronic Materials Engineering, Kwangwoon University, Seoul 01897, Korea; jmOH@kw.ac.kr (J.-M.O.); smkoo@kw.ac.kr (S.-M.K.)

**Keywords:** skutterudite, thermoelectric, atomic layer deposition, ZnO, SnO_2_

## Abstract

Nanostructuring is considered one of the key approaches to achieve highly efficient thermoelectric alloys by reducing thermal conductivity. In this study, we investigated the effect of oxide (ZnO and SnO_2_) nanolayers at the grain boundaries of polycrystalline In_0.2_Yb_0.1_Co_4_Sb_12_ skutterudites on their electrical and thermal transport properties. Skutterudite powders with oxide nanolayers were prepared by atomic layer deposition method, and the number of deposition cycles was varied to control the coating thickness. The coated powders were consolidated by spark plasma sintering. With increasing number of deposition cycle, the electrical conductivity gradually decreased, while the Seebeck coefficient changed insignificantly; this indicates that the carrier mobility decreased due to the oxide nanolayers. In contrast, the lattice thermal conductivity increased with an increase in the number of deposition cycles, demonstrating the reduction in phonon scattering by grain boundaries owing to the oxide nanolayers. Thus, we could easily control the thermoelectric properties of skutterudite materials through adjusting the oxide nanolayer by atomic layer deposition method.

## 1. Introduction

The regulation of thermal energy is a global issue and critical for reducing global warming, which causes climate change. Various renewable and sustainable-energy-related technologies, such as energy storage and conversion materials, have been researched as alternatives to fossil fuel energy technologies. The thermoelectric (TE) technology, which allows the direct conversion of thermal energy into electrical energy without generating any pollutant, is considered the key solution to control the abundant waste heat energy [1,2,3]. The conversion efficiency of TE power generation devices is determined by the thermoelectric dimensionless figure of merit *zT*, which can be calculated by the equation *zT* = *σ* ∙ *S*^2^ ∙ *T*/*κ*, where S is the Seebeck coefficient, *σ* is the electrical conductivity, *T* is the absolute temperature, and *κ* is the total thermal conductivity of the materials. The *κ* includes electronic (*κ_elec_*) and lattice (*κ_latt_*) contributions, with *κ_elec_* governed by Wiedemann–Frantz law (*κ_elec_* = *L* · *σ* · *T*, where *L* is Lorenz number) and *κ_latt_* determined by phonon scattering. Therefore, it is necessary to develop a facile method to enhance the electronic transport properties by reducing the thermal transport properties to achieve high TE performance [4,5,6].

Various TE materials have been studied in terms of their operating temperature [3]. Among them, skutterudite (SKD)-based compounds are considered one of the best candidates for medium-to-high-temperature applications owing to their good performance, stability, and mechanical properties [7,8]. Filled SKD materials with a composition of R*_x_*T_4_Pn_12_ (R = rare earth, actinide, alkali, and alkaline-earth metal; T = transition metal; Pn = pnictogen atom) composed of a cubic lattice of T_4_Pn_12_ and R elements as fillers (or rattlers), where the filler element tends to rattle, resulting in the effective scattering of lattice phonons, exhibit a low κ, representing the phonon-glass-electron-crystal model. Co_4_Sb_12_-based SKD materials show n-type conduction, and a variety of filler materials have been explored for Co_4_Sb_12_-based compounds [9,10,11,12,13,14,15]. Single elements such as In or Yb-filled n-type SKD materials have been investigated to effectively modulate the TE properties in terms of phonon scattering. He et al. reported a *zT* value of 1.2 for an In_0.25_Co_4_Sb_12_ n-type SKD material [9] and showed that the limit for In incorporation is ~0.22. A Yb-filled SKD has shown a high *zT* value of 1.5 for a high filling fraction of ~0.3 for Co_4_Sb_12_ [11]. Moreover, multiple filler elements are substantially used for broad-frequency phonon scattering, leading to reach high TE performance over 1.4 [13,14]. However, the Co_4_Sb_12_ mother matrix had a filling limitation for each filler element, which rendered the scattering of more phonons difficult. In addition to the control of filler composition, a different strategy such as grain boundary engineering is also required to enhance the phonon scattering of SKD materials to obtain high-performance TE materials.

Secondary phase incorporation is one of the promising technologies to enhance TE properties [15,16,17,18,19,20]. Much effort has been devoted to incorporating nanostructures such as nanoparticles or nanosheets into TE matrices. The atomic layer deposition (ALD) method, [21,22] which is typically used for thin film deposition, could be the best candidate for evaluating the effect of thin coating layer. Furthermore, the excellent conformality could be achieved even on the complex shaped substrate or powder materials [23]. The recent paper reveals that the introduction of ZnO on the grain boundary of Bi_0.4_Sb_1.6_Te_3_ significantly reduces the *κ_latt_* without deteriorating the electronic transport properties [18]. The effect of nanolayers at the grain boundaries introduced by ALD on the electronic and thermal transport properties of other TE materials needs to be studied.

In this study, we prepared SKD-based complex TE materials by the ALD technique and investigated the effects of nanolayer coating at the grain boundaries of SKD materials. We successfully synthesized ZnO- and SnO_2_-nanolayer-coated In_0.2_Yb_0.1_Co_4_Sb_12_ SKD powders by the ALD method and investigated the effect of the number of ALD cycles on the electronic and thermal transport properties.

## 2. Materials and Methods

An In_0.2_Yb_0.1_Co_4_Sb_12_ ingot was prepared by a typical solid-state reaction method. High-purity Co (99.998%, Alfa Aesar, Ward Hill, MA, USA), Sb (99.999%, Alfa Aesar, Ward Hill, MA, USA), In (99.999%, Alfa Aesar, Ward Hill, MA, USA), and Yb (99.9%, Alfa Aesar, Ward Hill, MA, USA) were weighed to obtain the stoichiometric composition of In_0.2_Yb_0.1_Co_4_Sb_12_ and charged into a carbon-coated quartz tube. The tube was vacuum-sealed to below 9.5 × 10^−3^ Torr, and the raw materials were melted using induction melting furnace for 20 min. The ingot was sealed again and annealed under 700 °C for one week. The ingot was pulverized using a high-energy ball mill (8000D, SPEX, Metuchen, NJ, USA) for 5 min. The resulting powder was sieved to obtain fine powder.

ZnO and SnO_2_ were deposited on the In_0.2_Yb_0.1_Co_4_Sb_12_ powder by ALD (iSAC Co. Ltd., iOVd100) in the temperature range of 150–350 °C using SnCl_4_ (99.995%, iChems Co. Ltd., Hwaseong, Korea) and (C_2_H_5_)_2_Zn (iChems Co. Ltd., Hwaseong, Korea), respectively, and H_2_O was used as an oxidant. Each deposition cycle consisted of a pulse of precursors, N_2_ purging, H_2_O pulse, and a second N_2_ purging process. Each purging process lasted for 20 s to allow the sufficient exhaustion of residual gases and physisorbed precursors. The precursor was supplied to the chamber with a 50 sccm flow of N_2_ gas. The deposition pressure was maintained as ~0.6 Torr, and 50 sccm N_2_ gas was continuously supplied to the chamber during the process. Each coating was applied to the In_0.2_Yb_0.1_Co_4_Sb_12_ powder with 1, 3, 5, and 100 ALD cycles.

Polycrystalline bulk samples were prepared by spark plasma sintering at 873 K for 5 min under 50 MPa in vacuum. Our bulk samples have the high density more than 96% of the theoretical density of SKD. The temperature-dependent *S* and *σ* parameters were measured from room temperature to 860 K (ZEM-3 Advanced-RIKO, Yokohama, Japan). The κ values of the samples were calculated by the equation *κ* = *ρ_s_* · *C_p_* ∙ *λ*, where *ρ_s_*, *C_p_*, and *λ* are the theoretical density, heat capacity, and thermal diffusivity, respectively. The λ values were measured using a laser flash installation (Laser Flash Analysis, LFA 467, Netzsch, Selb, Germany) from room temperature to 700 K. The microstructures of the ZnO and SnO_2_ coatings were analyzed by bright-field transmission electron microscopy (TEM, JEM-F200, JEOL, Tokyo, Japan).

## 3. Results and Discussion

**Figure 1:**
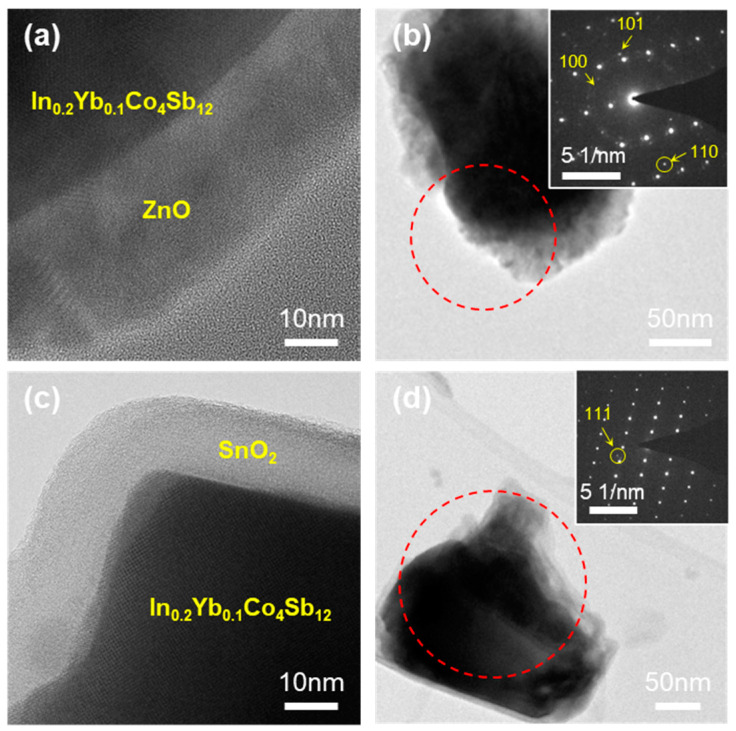
TEM images of ZnO- and SnO_2_-coated In_0.2_Yb_0.1_Co_4_Sb_12_ SKD powder prepared by ALD. (**a**) HRTEM image and (**b**) bright-field TEM image of ZnO-coated In_0.2_Yb_0.1_Co_4_Sb_12_ SKD powder (inset: SAED pattern of the area enclosed in dashed red circle in Figure 1b). (**c**) HRTEM image and (**d**) bright-field TEM image of SnO_2_-coated In_0.2_Yb_0.1_Co_4_Sb_12_ SKD powder (inset: SAED pattern of the area enclosed in dashed red circle in (**d**)).

Figure 1 shows the high-resolution TEM (HRTEM) images of the ZnO and SnO_2_-coated In_0.2_Yb_0.1_Co_4_Sb_12_ SKD powders after 100 cycles of ALD. As can be seen, the thickness of the ZnO layers after 100 ALD cycles is ~26 nm (Figure 1a), while that of the SnO_2_ layer after 100 ALD cycles is ~15 nm (Figure 1c). From the coating thicknesses, the ZnO and SnO_2_ ALD deposition rates were calculated as 2.60 Å/cycle and 1.50 Å/cycle, respectively. The polycrystalline ZnO and SnO_2_ layers were homogeneously coated on the In_0.2_Yb_0.1_Co_4_Sb_12_ SKD powder (Figure 1b,d). In addition, the selected area electron diffraction (SAED) patterns (inset images in Figure 1b,d) of the powder show concentric rings and well defined spots corresponding to the (100), (101), and (110) planes of hexagonal wurtzite ZnO and the (111) plane of orthorhombic SnO_2_, respectively. Thus, compared with other ALD methods reported in literature, our ALD method could synthesize homogeneous coating layers of ZnO and SnO_2_ on the In_0.2_Yb_0.1_Co_4_Sb_12_ SKD powder [18,19].

**Figure 2:**
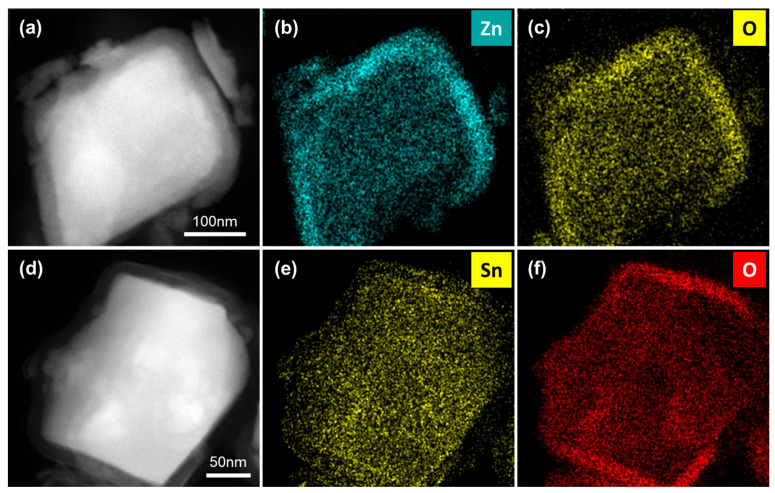
HAADF and EDS mapping images of ZnO- and SnO_2_-coated In_0.2_Yb_0.1_Co_4_Sb_12_ SKD powder obtained by ALD. (**a**) HAADF image, (**b**) Zn mapping image, and (**c**) O mapping image of ZnO-coated In_0.2_Yb_0.1_Co_4_Sb_12_ SKD powder. (**d**) HAADF image, (**e**) Zn mapping image, and (**f**) O mapping image of SnO_2_-coated In_0.2_Yb_0.1_Co_4_Sb_12_ SKD powder.

Figure 2 shows the high-angle annular dark field (HAADF) and energy dispersive X-ray spectroscopy (EDS) mapping images of the ZnO and SnO_2_-coated In_0.2_Yb_0.1_Co_4_Sb_12_ SKD powders after 100 ALD cycles. The bright region corresponds to the heavy elements of the In_0.2_Yb_0.1_Co_4_Sb_12_ matrix, whereas the dark region corresponds to the light elements of the ZnO or SnO_2_ coating layers (Figure 2a,d). The EDS mapping images of ZnO- (Figure 2b,c) and SnO_2_-coated In_0.2_Yb_0.1_Co_4_Sb_12_ SKD powders (Figure 2e,f) indicate the uniform coating of ZnO and SnO_2_ on each In_0.2_Yb_0.1_Co_4_Sb_12_ particle. This confirms that the facile ALD coating technology can produce a uniform coating of ZnO and SnO_2_ layers on the In_0.2_Yb_0.1_Co_4_Sb_12_ SKD powder. We expect that the homogeneous coating of oxide layer would affect the TE properties of the In_0.2_Yb_0.1_Co_4_Sb_12_ SKD material.

**Figure 3:**
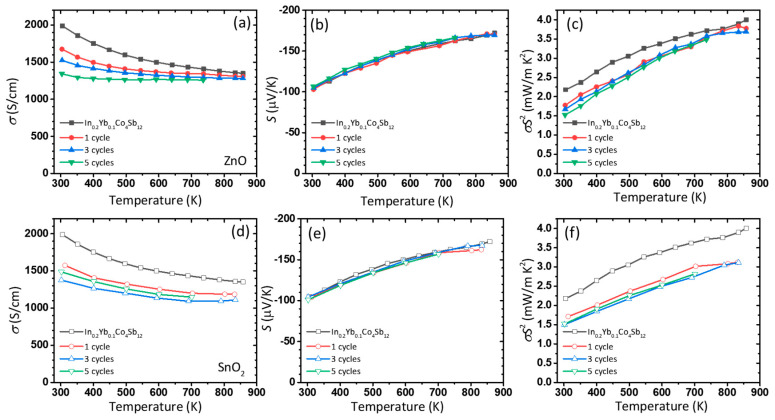
Electronic transport properties of ZnO- and SnO_2_-coated In_0.2_Yb_0.1_Co_4_Sb_12_ SKD samples prepared by ALD. (**a**,**d**) Electrical conductivity, (**b**,**e**) Seebeck coefficient, and (**c**,**f**) power factor as a function of temperature for ZnO (**a**–**c**) and SnO_2_ (**d**–**f**)-coated In_0.2_Yb_0.1_Co_4_Sb_12_ SKD samples.

Figure 3 shows the temperature-dependent electrical transport properties (*σ* and *S*) of pristine and ZnO and SnO_2_-coated In_0.2_Yb_0.1_Co_4_Sb_12_. The *σ* of pristine In_0.2_Yb_0.1_Co_4_Sb_12_ decreased with an increase in temperature, exhibiting a metallic conduction behavior (Figure 3a). However, the conduction became less metallic with the coating of a ZnO layer, and the *σ* of the ZnO-coated In_0.2_Yb_0.1_Co_4_Sb_12_ after 100 ALD cycles decreased with increasing temperature, exhibiting a semiconducting behavior. With increasing number of ALD cycles, the *σ* decreased over the entire temperature range because of the possible electron scattering by the oxide nanolayers formed at the grain boundaries. However, interestingly, the *S* changed only slightly even after 100 ALD cycles (Figure 3b). This indicates that the oxide coating only affects the carrier mobility. The *S* at room temperature varied from 100 µV/K to 112 µV/K, while the *σ* at room temperature decreased from 1988 S/cm for pristine In_0.2_Yb_0.1_Co_4_Sb_12_ to 410 S/cm for ZnO-coated In_0.2_Yb_0.1_Co_4_Sb_12_ after 100 ALD cycles. Therefore, the power factor is mainly governed by the *σ* in this case; hence, pristine In_0.2_Yb_0.1_Co_4_Sb_12_ exhibited the highest power factor of 2.18 mW/mK^2^ (Figure 3c,f). For SnO_2_ coatings, the temperature dependences of *σ* and *S* are similar to those observed for ZnO coatings, while SnO_2_-coated In_0.2_Yb_0.1_Co_4_Sb_12_ exhibited a slight metallic conduction behavior even after 100 ALD cycles (Figure 3d). The *S* did not change significantly with increasing number of ALD coating cycles (Figure 3e). Overall, the results indicate that the coating of ZnO and SnO_2_ is unfavorable for the electrical transport properties of In_0.2_Yb_0.1_Co_4_Sb_12_.

**Figure 4:**
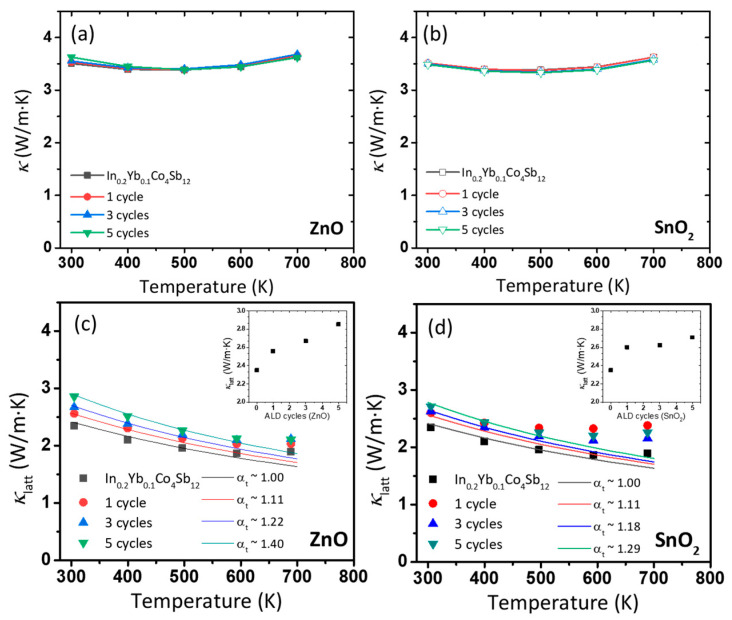
(**a**,**b**) Thermal conductivity and (**c**,**d**) lattice thermal conductivity of ZnO- and SnO_2_-coated In_0.2_Yb_0.1_Co_4_Sb_12_ SKD samples. The lines are calculated lattice thermal conductivity based on the Debye–Callaway model with different *α_t_* values. Insets of (**c**,**d**) show the lattice thermal conductivity at room temperature as a function of ALD cycles.

The measured *κ* and *κ_latt_* values are shown in Figure 4. As shown in Figure 4a,b, the samples exhibit similar *κ* values of 3.5 W/mK at room temperature. However, because of the decrease in *σ* due to the coatings, an increase in *κ_latt_* is expected despite the unchanged *κ*. Figure 4c,d show the calculated *κ_latt_* by deducting *κ_elec_* calculated by Wiedemann–Frantz law (*κ_elec_ = L* · *σ* · *T*), where *L* is the Lorenz number, which can be estimated by Equation (1) [24].

(1) $$L=1.5+\exp\left[-\frac{\left|S\right|}{116}\right]$$

The calculated *κ_latt_* for both the coatings increased with increasing number of ALD cycles. The insets of Figure 4c,d show the *κ_latt_* at room temperature. As can be seen, the *κ_latt_* gradually increased from 2.35 to 2.85 W/mK with an increase in the number of ZnO coating cycles to five.

The *κ_latt_* is represented by Debye–Callaway model as Equation (2):

(2) $$\kappa_{latt}=\frac{k_{B}}{2\pi^{2}\nu }{\left(\frac{k_{B}T}{\hbar }\right)}^{3}\int_{0}^{\theta_{a}/T}\frac{\tau_{tot}\left(z\right)z^{4}e^{z}}{{\left(e^{z}-1\right)}^{2}}dz,$$

where *ν*, *τ_tot_*, *k_B_*, *ħ*, *θa*, and *z* are the phonon group velocity, total phonon relaxation time, Boltzmann constant, reduced Planck’s constant, Debye temperature, and *ħω*/*k_B_T*, respectively. The *τ_tot_* in Equation (2) is estimated from the individual relaxation time for different defect structures (*τ_i_*) by Equation (3), while the inverse relaxation time for boundary phonon scattering (*τ_B_*^−1^) is expressed as Equation (4).

(3) $$\tau_{tot}{\left(z\right)}^{-1}=\Sigma_{i}\tau_{i}^{-1}\Sigma i$$

(4) $$\tau_{B}^{-1}=\frac{\nu }{a_{t}d}$$

Here, *α_t_* and *d* are the grain boundary transmission coefficient and grain size, respectively. Therefore, based on the underlying physics, changes in *α_t_* and *d* would lead to a modification of *κ_latt_*. In Equation (4), the *α_t_* was fitted to the experimental *κ_latt_* (symbols in Figure 4c,d) with assuming *α_t_* = 1.0 for In_0.2_Yb_0.1_Co_4_Sb_12_, while *d* is assumed as ~500 nm. For ZnO-coated samples, the *α_t_* becomes systematically larger to 1.11, 1.22 and 1.40 for. For SnO_2_ coated samples, the *α_t_* also increases systematically larger to 1.11, 1.18 and 1.29 for one, three, and five cycles. Although the fitting at high temperatures shows a discrepancy for SnO_2_-coated samples, the general trend of *κ_latt_* increase is maintained. The *κ* value generally affect both the intrinsic *κ* value of the certain material and the phonon scattering on the defect structures (e.g., grain boundaries). In our system, the intrinsically high *κ* values of ZnO and SnO_2_ (~50 and ~100 W/mK, respectively, at room temperature) seems to increase *α_t_*.

**Figure 5:**
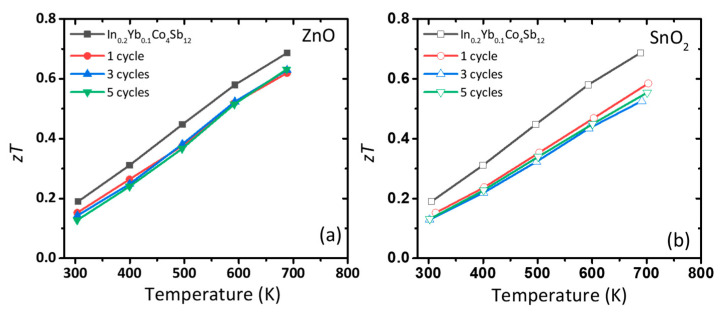
(**a**,**b**) Thermoelectric figure of merit (*zT*) of ZnO and SnO_2_-coated In_0.2_Yb_0.1_Co_4_Sb_12_ SKD samples.

The temperature-dependent *zT* values of ZnO- and SnO_2_-coated In_0.2_Yb_0.1_Co_4_Sb_12_ are shown in Figure 5. The *zT* values of pristine In_0.2_Yb_0.1_Co_4_Sb_12_ and that coated with ZnO with one, three, and five cycles of ALD are 0.69, 0.62, 0.63, and 0.63, respectively, at 700 K (Figure 5a). For SnO_2_-coated In_0.2_Yb_0.1_Co_4_Sb_12_, the *zT* decreased to 0.58, 0.53, and 0.55 after one, three and five ALD cycles, respectively (Figure 5b). The change in *zT* value by introducing ZnO and SnO_2_ nanolayer does not seem positive effect in our work due to the high intrinsic *κ* values of selected oxide materials. However, we suggest the ALD-coated structure to control the physical properties in SKD TE materials. We believe that the fine tuning of the composition of coating layer or condition of ALD process could realize the high TE performance in SKD materials.

## 4. Conclusions

We synthesized polycrystalline In_0.2_Yb_0.1_Co_4_Sb_12_ SKD with ZnO and SnO_2_ oxide nanolayers at grain boundaries by an ALD process. The ALD method enables the coating of oxide nanolayers on the surfaces of SKD powders. With increasing numbers of deposition cycles, the electrical conductivity gradually decreased, while the Seebeck coefficient changed only slightly. The oxide nanolayers formed at the grain boundaries scatter the carriers, thus decreasing the mobility. In contrast, the lattice thermal conductivity increased with increasing number of deposition cycles, demonstrating the reduction in phonon scattering by grain boundaries owing to the highly thermally conductive oxide nanolayers. Thus, our work does not show the positive effect of oxide nanolayer coating on TE performance but gives a facile strategy for controlling electrical/thermal transport properties on a simple ALD process.
